# Identifying high-risk individuals for lung cancer screening: Going beyond NLST criteria

**DOI:** 10.1371/journal.pone.0195441

**Published:** 2018-04-05

**Authors:** Marcela Fu, Noémie Travier, Juan Carlos Martín-Sánchez, Jose M. Martínez-Sánchez, Carmen Vidal, Montse Garcia, on behalf of the LUCAPREV research group

**Affiliations:** 1 Tobacco Control Unit, Cancer Prevention and Control Programme, Catalan Institute of Oncology-IDIBELL, L'Hospitalet de Llobregat, Llobregat, Spain; 2 Cancer Screening Unit, Cancer Prevention and Control Programme, Catalan Institute of Oncology-IDIBELL, L’Hospitalet de Llobregat, Llobregat, Spain; 3 Group of Evaluation of Health Determinants and Health Policies, Universitat Internacional de Catalunya, Sant Cugat del Vallès, Spain; National Health Research Institutes, TAIWAN

## Abstract

**Background:**

There are two main types of strategies to identify target population for lung cancer screening: 1) strategies based on age and cumulative smoking criteria, 2) risk prediction models allowing the calculation of an individual risk. The objective of this study was to compare different strategies to identify the proportion of the Spanish population at high risk of developing lung cancer, susceptible to be included in a lung cancer screening programme.

**Methods:**

Cross-sectional study. We used the data of the Spanish National Interview Health Survey (ENSE) of 2011–2012 (21,006 individuals) to estimate the proportion of participants at high risk of developing lung cancer. This estimation was performed using the U.S. national lung screening trial (NLST) criteria and a 6-year prediction model (PLCO_m2012_), both independently and in combination.

**Results:**

The prevalence of individuals at high risk of developing lung cancer according to the NLST criteria was 4.9% (7.9% for men, 2.4% for women). Among the 1,034 subjects who met the NLST criteria, 533 (427 men and 106 women) had a 6-year lung cancer risk ≥2.0%. The combination of these two selection strategies showed that 2.5% of the Spanish population had a high risk of developing lung cancer. However, this selection process did not take into account different groups of subjects <75 years old having an individual risk of lung cancer ≥2%, such as heavy smokers <55 years old who were long-time former smokers, and ever-smokers having smoked <30 pack-years with other risk factors.

**Conclusions:**

Further research is needed to determine which selection strategy achieves a higher benefit/harm ratio and to assess other prevention strategies for individuals with elevated risk for lung cancer but who do not meet the screening eligibility criteria.

## Introduction

Lung cancer is the most frequent of all cancers diagnosed in Europe and also the most common cause of cancer-related death [1], with an age-standardised 5-year survival of 13% for adult patients with cancer diagnosed in 2000–2007 [2].

The implementation of a lung cancer screening programme at population level is controversial as lung cancer screening has both benefits and harms [3,4] and previous trials have shown inconsistent results. The national lung screening trial (NLST) in the United States found that a lung cancer screening programme using annual computed tomography (CT) at low dose for three years in high-risk ever-smokers may reduce lung cancer mortality by 20% compared with conventional thoracic radiography [5]. In this trial, high-risk ever-smokers were defined as ever-smokers aged 55–74 years old, having smoked ≥30 pack-years and with ≤15 years since cessation for quitters. On the other hand, in Europe, the Danish lung cancer screening trial (DLCST) compared low dose CT screening with no screening in a different group of high-risk ever-smokers (50–70 years old, having smoked ≥20 pack-years, age at cessation >50 years old and quitting time <10 years for former smokers) and did not find any significant differences in lung cancer mortality [6]. Another large European trial, the Dutch-Belgian randomised lung cancer screening trial (NELSON), is actually ongoing. This trial aims at comparing low dose CT screening with no screening in ever-smokers aged 50–75 years, who smoked >15 cigarettes per day for >25 years or >10 cigarettes for >30 years, and were still smoking or had quit ≤10 years before recruitment [7].

Individuals at high risk of developing lung cancer may benefit from early detection through screening based on low-dose CT. However, because low-dose CT screening has non-negligible adverse effects (radiation exposure, false positives and overdiagnosis), identifying the most appropriate target population is essential to maximise screening benefits and minimize adverse effects. To help define the target population for lung cancer screening, some models allowing the calculation of the individual risk of developing lung cancer have previously been published [8]. These models take into account important risk factors, such personal and familiar disease history and other relevant aspects of smoking, including smoking duration or intensity [9], whereas the screening eligibility criteria used in the aforementioned clinical trials are only based on age and the amount smoked in pack-years.

The objective of the present paper is to compare different strategies that could be used to identify the proportion of the Spanish population at high risk of developing lung cancer, susceptible to be included in a lung cancer screening programme.

## Methods

Although the analysis could have been performed without going through a research ethics committee, as the data involved were de-identified and available for public use, we obtained the approval of a Clinical Research Ethics Committee of the Bellvitge University Hospital (ref PR249/16), as this analysis is part of a broader project entitled “Cost-effectiveness and budget impact analysis of three preventive strategies in lung cancer".

### Study design and subjects

This is a cross-sectional analysis of the data from the Spanish National Health Survey (*Encuesta Nacional de Salud de España*, ENSE), a cross-sectional survey on subjects ≥15 years old, representative of the non-institutionalized Spanish population.

This survey is conducted every five years and gathers health related information at national level. Detailed information on the ENSE methodology is available on the website of the Spanish Ministry of Health (www.msssi.gob.es/en/estadEstudios/estadisticas/encuestaNacional/ense.htm).

Briefly, survey participants were selected by means of probabilistic multistage sampling in order to obtain representative data at regional and national level. The sampling method consisted of a multistage cluster, where primary units were census tracts, secondary units were households and the tertiary units (individuals) were selected from the description of household members at the time of the interview. A sex and age-stratified sampling scheme have been used for this survey.

The latest ENSE data available were collected in 2011–2012, they include information on 21,508 individuals, 21,006 having complete smoking history information. For the present analysis, no consent statement from participants was necessary, as all microdata are anonymised and openly available on the aforementioned website.

### Variables and analysis

The data of the ENSE survey were used to estimate the proportion of individuals at high risk of developing lung cancer in the general population and among ever-smokers. High-risk participants were first defined using the NLST and NELSON trials criteria, based on age and cumulative smoking exposure. The proportion of participants at high risk of developing lung cancer obtained from the ENSE sample was then extrapolated into an absolute figure for the Spanish population, using the latest available population census data of 2014 from the National Statistics Institute (www.ine.es). Then, we estimated the 6-year individual risk of developing lung cancer of current and former smokers from the ENSE survey using the model developed in the context of the prostate, lung, colorectal and ovarian screening trial (PLCO trial) [10]. The validated 6-year prediction model for ever-smokers developed by Tammemägi et al. (PLCO_m2012_) includes age, race/ethnicity, education, body mass index, personal history of cancer, family history of lung cancer, chronic obstructive pulmonary disease (COPD), smoking status, tobacco consumption, smoking duration and time since quitting [11]. In the present analysis we did not include the family history of lung cancer and ethnicity variables, as this information was not available in the ENSE survey, and therefore assumed there was no risk due to family history of lung cancer and that all population was Caucasian. Instead of the 6-category education variable of the PLCO_m2012_ model, we used a variable indicating the Spanish socioeconomic status of the head of household [12]. This variable includes the following six categories: professions associated to postgraduate university degrees; professions associated to graduate university degrees and qualified technicians; administrative employees and professionals, personal service and self-employed workers, and supervisors of manual workers; skilled and semi-skilled manual workers; unskilled workers. We described the distribution of the individual 6-year risk in quintiles of risk and also identified individuals with the following risk thresholds: ≥1.51% [11], ≥2.00% [13] and ≥5.00% [14].

Tammemägi et al. [11] found that ≥1.51% level of risk, calculated with PLCO_m2012_, yielded a mortality benefit for low-dose CT screening in the NLST and the number needed to screen to prevent one lung cancer death would be reduced from 320 to 255. We also considered a 2% threshold risk used in a study aimed to validate the performance of PLCO_m2012_ in predicting lung cancer outcomes in a cohort of Australian smokers. The study showed that it performed better than the NLST [13]. Finally, we also considered the upper threshold (>5.00%) used in the Liverpool Lung Project Risk Prediction Model for lung cancer incidence (LLP_v2_), in which individuals whose 5-year predicted absolute risk was above 5.00% were designated as “high-risk” group [14]. This threshold corresponded to the value for the 20% of predicted absolute risk in the general Liverpool population. This risk algorithm has been used as the basis for risk assessment in the UK Lung Cancer Screening Trial [14].

Finally, we described lung cancer risk factors [15] of NLST and ENSE participants at high risk of developing lung cancer. For ENSE participants, three definitions were used to identify high-risk participants: 1) individuals meeting NLST criteria, 2) individuals meeting NLST criteria having a 6-year lung cancer risk of 2% or higher, and 3) individuals younger than 75 years having a 6-year lung cancer risk of 2% or higher, irrespective of NLST criteria.

## Results

In the ENSE survey, the proportion of individuals at high risk of developing lung cancer was 6.6% (95% CI: 6.2%; 6.9%) according to the NELSON criteria and 4.9% (95% CI: 4.6%; 5.2%) according to the NLST criteria (Table 1). The extrapolation of these percentages into absolute figures shows that in Spain 2,653,744 individuals (1,862,034 men and 791,710 women) would be considered at high risk of developing lung cancer if the NELSON criteria were applied. This figure would went down to 2,003,483 individuals (1,523,120 men and 480,364 women) when the NLST criteria were used.

**Table 1:** Individuals (%) at high risk of developing lung cancer using the ENSE survey of 2011–2012 and extrapolation into absolute figures for the target population at national level*.

	Population at risk in the ENSE sample			Target population at national level*		
	N	NELSON criteria^†^	NLST criteria^‡^	Total population	NELSON criteria^†^	NLST criteria^‡^
		% (95% CI)	% (95% CI)	n	n	N
General population**						
All	21,006	6.6 (6.2; 6.9)	4.9 (4.6; 5.2)	39,441,665	2,653,744	2,003,483
Men	9,648	9.7 (9.1; 10.3)	7.9 (7.4; 8.5)	19,234,370	1,862,034	1,523,120
Women	11,358	3.9 (3.6; 4.3)	2.4 (2.1; 2.7)	20,207,295	791,710	480,364
Ever-smoker population***						
All	9,496	14.5 (13.8; 15.2)	10.9 (10.3; 11.5)	18,122,937	2,653,744	2,003,483
Men	5,727	16.3 (15.4; 17.3)	13.3 (12.5; 14.2)	11,417,417	1,862,034	1,523,120
Women	3,769	11.8 (10.8; 12.8)	7.2 (6.3; 8.0)	6,705,520	791,710	480,364

* Target population using Spanish population census data of 2014 (www.ine.es).

** All population was used in the denominator for the computation of the prevalence.

*** Ever-smoker population was used in the denominator for the computation of the prevalence.

^**†**^ NELSON eligibility criteria: Age: 50–74 years. Smoking history: >15 cigarettes/day during >25 years; or >10 cigarettes/day during >30 years; if former smokers, quitting time ≤10 years. Trial registration number: ISRCTN63545820.

^**‡**^ NLST eligibility criteria: Age: 55–74 years. Smoking history: pack-years ≥30 years; if former smokers, quitting time ≤15 years. Trial registration number: NCT00047385

CI: confidence interval.

Table 2 shows the distribution of the 6-year risk of developing lung cancer of ENSE participants who fulfilled the NLST criteria. This table shows that 72% of individuals who met the NLST criteria exceeded the ≥1.51% risk threshold. More than a half of current and former smokers (56%) who had quit for less than 15 years, were aged 55–74 years old and had smoked ≥30 pack-years, had a risk of developing lung cancer above 2%. Men showed a higher risk than women; the median risk of developing lung cancer was 2.3% in men and 1.9% in women. Survey participants who had a risk of developing lung cancer ≥5% represent 15.3% of men and 6.7% of women. When percentages were applied to the Spanish population, we estimated that 2.5% of the Spanish population (1,039,860 individuals; 851,272 men and 188,587 women) may have a risk of developing lung cancer ≥2% (results not shown).

**Table 2:** Distribution of the risk of developing lung cancer of Spanish ever-smokers who fulfilled the NLST criteria based on a 6-year risk model*.

	ENSE sample fulfilling NLST criteria		
	Overall (n = 1,034) **	Men (n = 764) **	Women (n = 270) **
Mean	2.91	3.12	2.26
Median	2.18	2.31	1.86
Quintile 1	0.55–1.30	0.55–1.36	0.56–1.21
Quintile 2	1.30–1.87	1.36–2.00	1.22–1.64
Quintile 3	1.87–2.64	2.00–2.84	1.65–2.16
Quintile 4	2.64–3.89	2.85–4.37	2.16–3.09
Quintile 5	3.90–21.98	4.38–21.98	3.09–9.14
Individuals with risks ≥1.51%, n (%)	685 (72.0)	535 (74.9)	150 (63.0)
Individuals with risks ≥2.00%, n (%)	533 (56.0)	427 (59.8)	106 (44.5)
Individuals with risks >5.00%, n (%)	125 (13.1)	109 (15.3)	16 (6.7)

* Based on the PLCO_m2012_ model, we included the following variables: age, socioeconomic status, body mass index, COPD, personal history of cancer, smoking status, tobacco consumption, smoking duration and years of abstinence.

** Because of missing values for some of the variables used in the PLCO_m2012_ model, the risk of developing lung cancer could only be calculated for 952 (714 men and 238 women) ENSE participants fulfilling NLST criteria.

Table 3 shows smoking-related and other lung cancer risk factors among the populations of the NLST trial and ENSE survey to which we applied the NLST criteria. The major differences observed between these two populations were: a lower proportion of women in the Spanish survey (26.1% vs 41.0% in the NLST trial) and a higher proportion of people smoking 20 or more cigarettes per day (87.2% vs. 52.5% in the NLST trial). Table 3 also shows the characteristics of the ENSE participants meeting the NLST criteria and having a risk of developing lung cancer ≥2%, calculated using the PLCO_m2012_ risk prediction model. This subpopulation at higher risk of developing lung cancer included a higher proportion of subjects who were older, had a diagnosis of COPD, and had smoked 40 or more pack-years.

**Table 3:** Distribution of selected lung cancer risk factors among the NLST and ENSE populations at high risk of developing lung cancer*.

	NLST trial** (n = 53,158)		ENSE sample with NLST criteria (n = 1,034)		ENSE sample with NLST criteria and 6-year lung cancer risk ≥2%* (n = 533)	
	N	% (95% CI)	N	% (95% CI)	N	% (95% CI)
***Sex***						
Men	31,365	59.0 (58.6; 59.4)	764	73.9 (71.2; 76.6)	427	80.1 (76.7; 83.5)
Women	21,793	41.0 (40.6; 41.4)	270	26.1 (23.4; 28.8)	106	19.9 (16.5; 23.3)
***Age (years)***						
55–59	22,705	42.7 (42.3; 43.1)	366	35.4 (32.5; 38.3)	70	13.1 (10.3; 16.0)
60–64	16,288	30.6 (30.3; 31.0)	308	29.8 (27.0; 32.6)	167	31.3 (27.4; 35.3)
65–69	9,477	17.8 (17.5; 18.2)	228	22.1 (19.5; 24.6)	185	34.7 (30.7; 38.8)
70–74	4,681	8.8 (8.6; 9.0)	132	12.8 (10.7; 14.8)	111	20.8 (17.4; 24.3)
***Body mass index***						
Underweight	458	0.9 (0.8; 0.9)	6	0.6 (0.1; 1.1)	6	1.1 (0.2; 2.0)
Normal	14,804	27.9 (27.6; 28.3)	280	29.0 (26.2; 31.9)	165	31.0 (27.0; 34.9)
Overweight	22.722	42.9 (42.5; 43.3)	456	47.3 (44.1; 50.4)	253	47.5 (43.2; 51.7)
Obese	14,985	28.3 (27.9; 28.7)	223	23.1 (20.4; 25.8)	109	20.5 (17.0; 23.9)
***Smoking status***						
Current smoker	25,585	48.1 (47.7; 48.6)	474	45.8 (42.8; 48.9)	264	49.5 (45.3; 53.8)
Former smoker	27.573	51.9 (51.5; 52.3)	560	54.2 (51.1; 57.2)	269	50.5 (46.2; 54.7)
***Cigarettes per day***						
10–19	25,261	47.5 (47.1; 47.9)	133	12.9 (10.8; 14.9)	67	12.6 (9.8; 15.4)
20–29	14,492	27.3 (26.9; 27.6)	493	47.7 (44.6; 50.7)	218	40.9 (36.7; 45.1)
30–39	9,625	18.1 (17.8; 18.4)	149	14.4 (12.3; 16.6)	82	15.4 (12.3; 18.4)
40–59	3,357	6.3 (6.1; 6.5)	192	18.6 (16.2; 20.9)	115	21.6 (18.1; 25.1)
60–79	349	0.7 (0.6; 0.7)	54	5.2 (3.9; 6.6)	39	7.3 (5.1; 9.5)
≥80	74	0.1 (0.1; 0.2)	13	1.3 (0.6; 1.9)	12	2.3 (1.0; 3.5)
***Years of smoking***						
<30	5,709	10.7(10.5–11.0)	59	5.7 (4.3; 7.1)	11	2.1 (0.9; 3.3)
30–39	23,130	43.5 (43.1–43.9)	317	30.7 (27.8; 33.5)	81	15.2 (12.1; 18.2)
40–49	24,319^†^	45.7 (45.3–46.2)	494	47.8 (44.7; 50.8)	293	55.0 (50.7; 59.2)
≥50			164	15.9 (13.6; 18.1)	148	27.8 (24.0; 31.6)
***Pack-years***						
30–39	13,662	25.7 (25.3; 26.1)	289	27.9 (25.2; 30.7)	71	13.3 (10.4; 16.2)
40–49	14,099	26.5 (26.2; 26.9)	274	26.5 (23.8; 29.2)	132	24.8 (21.1; 28.4)
50–59	7,358	13.8 (13.6; 14.1)	124	12.0 (10.0; 14.0)	80	15.0 (12.0; 18.0)
≥60	18,018	33.9 (33.5; 34.3)	347	33.6 (30.7; 36.4)	250	46.9 (42.7; 51.1)
***Years of abstinence for former smokers***						
<5	11,301	41.4 (40.9; 42.0)	168	30.0 (26.2; 33.8)	97	36.1 (30.3; 41.8)
5–9	7,732	28.4 (27.8; 28.9)	172	30.7 (26.9; 34.5)	77	28.6 (23.2; 34.0)
10–14	8.239	30.2 (29.7; 30.8)	220	39.3 (35.2; 43.3)	95	35.3 (29.6; 41.0)
***COPD diagnosis***						
No	48,938	92.3 (92.1; 92.5)	901	87.1 (85.1; 89.2)	433	81.2 (77.9; 84.6)
Yes	4,085	7.7 (7.5; 7.9)	133	12.9 (10.8; 14.9)	100	18.8 (15.4; 22.1)

* Based on the PLCO_2012_ model that includes: age, socioeconomic status, body mass index, COPD, personal history of cancer, smoking status, tobacco consumption, smoking duration and years of abstinence. The NLST trial’s data was extracted from Katki et al. [15] and Kovalchik et al. [16].

^†^ Information available for the population of the NLST trial: ≥40 years smoked.

** The NLST trial included ever-smokers aged 55–74 years old, having smoked ≥30 pack-years and ≤15 years since cessation for quitters.

Note: Some variables do not sum up the total due to some missing values.

**Fig 1:**
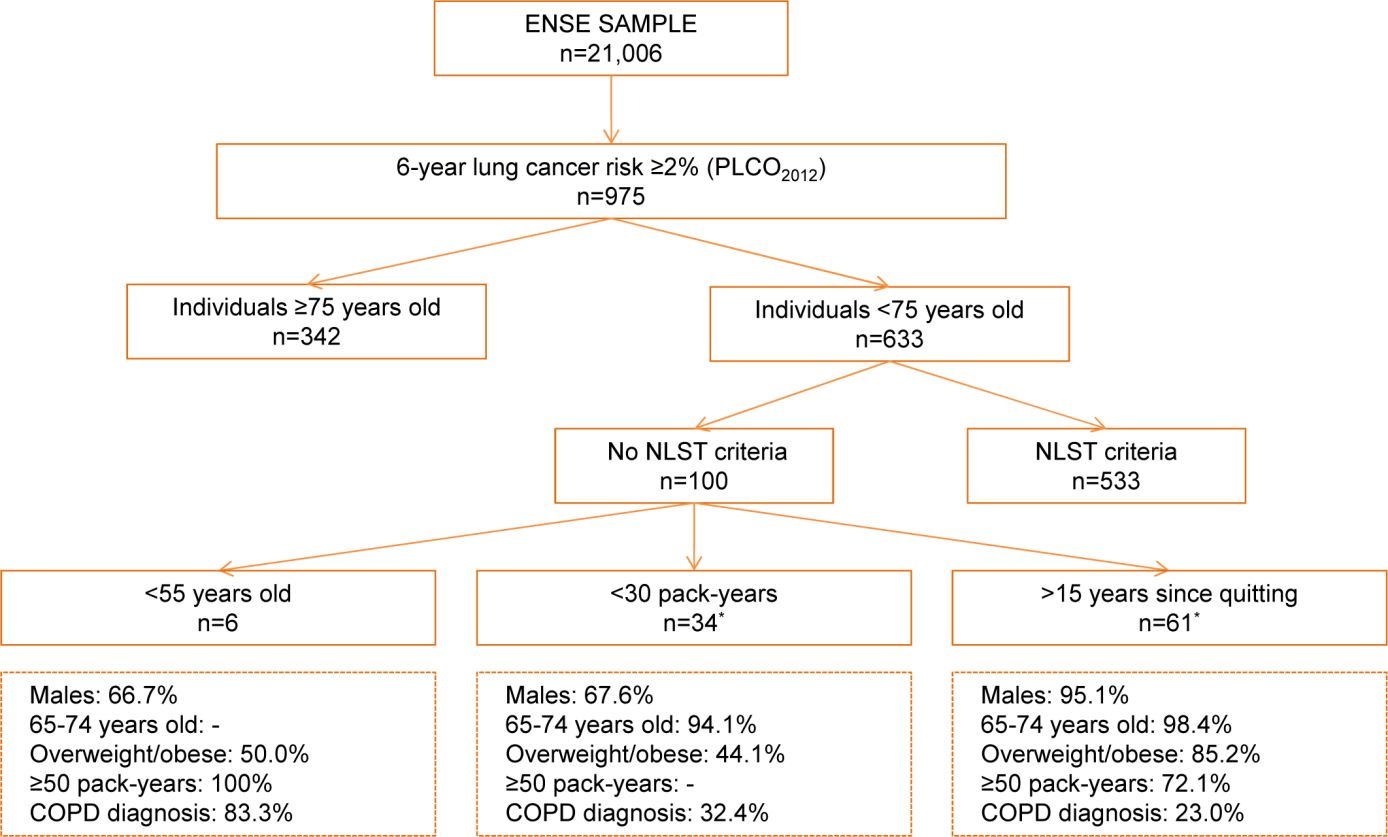
Distribution of ENSE participants with a 6-year lung cancer risk ≥2% who did not meet the NLST criteria. * One individual was a former smoker for more than 15 years and used to smoke <30 pack-years. Thus, the total sums up 101, not 100.

Finally, Fig 1 describes ENSE participants with a high risk of developing lung cancer (6-year risk ≥2% according to the PLCO_m2012_ model) who would not be screened if the NLST criteria were used to define the target population in the Spanish population. Among the 975 subjects having a 6-year risk ≥2%, 342 did not meet the NLST criteria because they were 75 years old or older. The remaining group of 100 subjects who did not fulfil the NLST criteria can be divided into three main groups. First, a group represents 6% of this subpopulation that includes males <55 years old, often underweight, with extremely high cigarette consumption. Second, a group representing 34% of the subpopulation that includes both men and women who smoked less than 30 pack-years but had other risk factors, such a COPD diagnosis. Third, a group representing 61% of the subpopulation, that includes older males, often overweight or obese, who stopped smoking after having smoked for many years.

Regarding the lung cancer risk of never-smokers, from 9,630 never-smokers, only 17 (0.18%) showed ≥2% risk of dying from lung cancer within 6 years.

## Discussion

The present study showed that an important part of the Spanish population may be at high risk of developing lung cancer and could possibly benefit from screening. The application of the inclusion criteria used by the NLST trial to a national health survey in Spain indicated that 4.9% of the survey participants were at high risk of developing lung cancer. The smoking characteristics of the participants of the Spanish survey were significantly different from those of the NLST trial. Both samples showed a similar distribution of the pack-years variable, but the participants of the Spanish survey smoked more cigarettes per day than those of the NLST trial. This finding stressed the importance of defining the lung cancer screening criteria that would best fit the specific characteristics and needs of the Spanish population.

Previous studies have shown that individuals at high risk of developing lung cancer may benefit from early detection based on low-dose CT screening [5]. Kovalchik et al. evaluated whether low-dose CT screening benefits and harms varied according to the distribution of the lung cancer risk; they found that low-dose CT screening prevented the greatest number of deaths from lung cancer among participants who were estimated to be at the highest risk of developing lung cancer [16]. Conversely, they also showed that low-dose CT screening prevented very few deaths among those estimated to be at the lowest risk. Therefore, identifying the most appropriate target population is essential to maximize screening benefits and minimize adverse effects [17–19].

Several authors have previously tried to define strategies allowing the identification of target populations for lung cancer screening. The two main types of strategies previously defined were based on age and cumulative smoking exposure criteria on one hand (as in NELSON and NLST trials) and on risk prediction models allowing the calculation of an individual risk on the other hand [15,16]. Strategies using age and cumulative smoking exposure criteria are easier to implement; however, comparative studies have been shown they might be inferior to strategies involving individual risk calculation [8,9,20]. For this reason, we decided to use these two types of strategies both independently and in combination to identify which way of selecting individuals for lung cancer screening would best fit our population.

The age and cumulative smoking exposure criteria used in the present study were those of the large NLST trial, that showed a 20% decrease in mortality from lung cancer when low-dose CT was compared with conventional thoracic radiography [5]. The risk prediction model used was the PLCO_m2012_ model with a cut-off point of 2%. This threshold was chosen as a recent study showed it performed better than the NLST, with superior sensitivity and specificity and had higher sensitivity than the U.S. Preventive Services Task Force risk criteria [21] with no loss in specificity [13].

However, we also calculated the proportion of individuals using a cut-off point of 1.51% risk, but almost 3 out of 4 individuals who met NLST criteria exceeded this level of risk. On the other hand, only 13.1% of individuals achieved the upper threshold (>5.0%). When resources are limited and/or the intervention carried serious adverse effects, selecting a very high-risk population is required to have a strong benefit-harm balance. The use of a conservative threshold is important, because previous studies have shown that low-dose CT screening can lead to harm [22]. Also, the effect of restricting screening to a subpopulation of high-risk individuals will reduce the cost of screening programmes at the expense of missing a proportion of lung cancers in individuals below the cut-off. This high-risk strategy aims to help individuals with the greatest need of, and the potential to benefit from early detection. Such stratification, mainly based on costs, available resources and public health impact of screening, implies the difficult decision of where to place the cut-off [19].

When we applied the NLST criteria along with the PLCO_m2012_ lung cancer risk of ≥2% to the ENSE sample, we found out that 56.0% of the participants meeting the NLST criteria had a 6-year risk ≥2%, representing 2.5% of the overall ENSE survey sample. According to these figures, we estimated that in Spain 1,039,860 individuals (851,272 men and 188,587 women) were at high risk of developing lung cancer and could possibly get some benefit from being screened. However, we also found that the combination of these two strategies would leave out four groups of subjects of very different characteristics. The first group, which included individuals ≥75 years old, is generally excluded from screening as mortality prevention due to competing risks of death is likely to be less than for younger counterparts and may not fit a curative treatment (surgery). In addition, adverse effects derived from the follow-up of lung nodules with invasive diagnostic procedures are higher among the elderly.

The other three groups of individuals <75 years old with an individual risk of lung cancer ≥2% included: (i) heavy smokers <55 years old, (ii) long-time former smokers with a quitting time >15 years, and (iii) ever-smokers having smoked <30 pack-years but having other risk factors, such as obesity or COPD diagnosis. It is not clear whether these three groups should be offered low-dose CT screening; however, their high risk of developing lung cancer should be taken into account and they should be the target of strategies designed to reduce and/or monitor their risk on a more individual basis [23]. We observed that different eligibility criteria lead to selection of partially non-overlapping population. Further research is needed to determine which selection strategy achieves a higher benefit/harm ratio and to assess other prevention strategies for individuals with elevated risk for lung cancer but who do not met the screening eligibility criteria.

The approach used in the present analysis, that highlighted disparities between two different ways of selecting the target population for lung cancer screening, corroborates the idea that ‘one size may not fit all’ and that screening is likely to progressively become more closely tailored to the actual level of risk of each individual [24].

Regarding lung cancer risk among never-smokers, we found that only 0.2% of them had ≥2% risk of developing lung cancer over a 6-year period. Ten Haaf and de Koning conducted a microsimulation study to assess if never-smokers at elevated risk could be eligible for lung cancer screening and if they may benefit from it. Their conclusion was that for most never-smokers lung cancer screening is not beneficial [25].

Some limitations to this study deserve consideration. We could not include the family history of lung cancer or race/ethnicity as additional factors in the identification of ever-smokers at highest risk of developing lung cancer, as this information was not gathered by the survey; nevertheless, ethnicity is not such a relevant variable in Spain (high proportion of Caucasian: 93%-95%) [26], as it is in the United States.

On the other hand, this study is the first one that estimates the proportion of individuals at high risk of developing lung cancer in Spain, that may benefit from lung cancer screening, using both age and cumulative smoking exposure criteria and a risk model allowing individual risk calculation.

In conclusion, the present study estimated that 2.5% of the Spanish population (1,039,860 individuals) is at high risk of developing lung cancer using the NLST criteria and the ≥2% risk threshold from PLCO_m2012_ combined, and could therefore be the target population for a lung cancer screening programme. However, the selection strategy applied systematically may have failed to identify specific subgroups of subjects, which could also possibly benefit from programmes designed to reduce and/or monitor their lung cancer risk. These findings showed that lung cancer screening might benefit from a selection of the target population more closely tailored to the level of risk of each individual.
